# Impact of Surgical Resection and Reasons for Poor Prognosis of Pelvic Osteosarcoma Based on the Bone Tumor Registry in Japan

**DOI:** 10.3390/cancers13133320

**Published:** 2021-07-01

**Authors:** Satoshi Takenaka, Hironari Tamiya, Toru Wakamatsu, Sho Nakai, Yoshinori Imura, Hidetatsu Outani, Toshinari Yagi, Akira Kawai

**Affiliations:** 1 Musculoskeletal Oncology Service, Osaka International Cancer Institute, Osaka 541-8567, Japan; hironari.tamiya@oici.jp (H.T.); wakamatu-to@mc.pref.osaka.jp (T.W.); s.nakai.0925@gmail.com (S.N.); yagi-to@mc.pref.osaka.jp (T.Y.); 2 Department of Orthopedic Surgery, Osaka University Graduate School of Medicine, Suita, Osaka 565-0871, Japan; y.imura@mc.pref.osaka.jp (Y.I.); h-otani@ort.med.osaka-u.ac.jp (H.O.); 3 Musculoskeletal Oncology and Rehabilitation Medicine, National Cancer Center, Tokyo 104-0045, Japan; akawai@ncc.go.jp

**Keywords:** pelvic osteosarcoma, surgical resection, prognosis, propensity score

## Abstract

**Simple Summary:**

Pelvic osteosarcoma has a poor prognosis compared to osteosarcomas in other locations, and the reasons for this remains unknown. Surgical resection of pelvic osteosarcoma is technically demanding and often results in dysfunction and complications. In this study, we investigated the reasons underlying the poor prognosis of pelvic osteosarcoma by comparing it to femoral osteosarcoma using data from the Bone Tumor Registry in Japan. We used propensity score analysis to determine whether surgical resection of pelvic osteosarcoma improved its prognosis. We demonstrated that pelvic osteosarcoma had a poor prognosis because it occurred more often in the elderly, often had a larger tumor size, and had metastasis at presentation more often in comparison to femoral osteosarcoma. These three factors were also associated with the non-surgical treatment of pelvic osteosarcoma, which also led to a poor outcome. The overall survival rate was only comparable in pelvic osteosarcoma and femoral osteosarcoma in cases treated with surgical resection. Propensity score analysis revealed that surgical treatment improved the prognosis of pelvic osteosarcoma. As such, we propose that surgical resection should be considered based on tumor stage and patient age in order to improve the prognosis of pelvic osteosarcoma.

**Abstract:**

Pelvic osteosarcoma has a poor prognosis compared to osteosarcomas in other locations, and the reasons for this remain unknown. Surgical resection of pelvic osteosarcoma is technically demanding and often results in dysfunction and complications. In this study, we investigated the reasons underlying the poor prognosis of pelvic osteosarcoma by comparing it to femoral osteosarcoma using data from the Bone Tumor Registry in Japan. We used propensity score analysis to determine whether surgical resection of pelvic osteosarcoma improved its prognosis. We demonstrated that pelvic osteosarcoma had a poor prognosis because it occurred more often in the elderly, often had larger tumor size, and had metastasis at presentation more often in comparison to femoral osteosarcoma. These three factors were also associated with the non-surgical treatment of pelvic osteosarcoma, which also led to a poor outcome. The overall survival rate was only comparable in pelvic osteosarcoma and femoral osteosarcoma in cases treated with surgical resection. Propensity score analysis revealed that surgical treatment improved the prognosis of pelvic osteosarcoma. As such, we propose that surgical resection should be considered based on tumor stage and patient age in order to improve the prognosis of pelvic osteosarcoma.

## 1. Introduction

Osteosarcoma is a malignant bone tumor that frequently occurs around the knee in adolescence. Advances in chemotherapy and surgical treatment for osteosarcoma have resulted in an expected 5-year survival rate of 60 to 80% [1,2,3,4]. In contrast, osteosarcoma of the pelvis, which accounts for less than 10% of all osteosarcomas, has a 5-year survival rate of 18 to 38% [5,6,7,8,9]. The reasons for the poor prognosis of pelvic osteosarcoma may be that it is often a secondary osteosarcoma, chondroblastic osteosarcoma, or that it has metastasis and large tumor size at presentation. Few studies have focused on why the prognosis of the pelvic osteosarcoma is poor.

Surgical resection is one of the main treatments for osteosarcoma, although there have been no randomized controlled studies on it. The anatomy of the pelvis makes it difficult to excise a tumor with adequate margins. Surgical treatments for pelvic osteosarcoma have been reported to lead to severe dysfunction and high complication rates [10,11,12]. From this background, surgeons are reluctant to perform surgical treatment for pelvic osteosarcoma.

In this study, we investigated the reasons for the poor prognosis of pelvic osteosarcoma by comparing it with femoral osteosarcoma. We also used propensity score analysis to examine whether surgical resection of pelvic osteosarcoma improved its prognosis. Finally, we assessed the indication of surgical resection of pelvic osteosarcoma. We used data from the Bone Tumor Registry in Japan, which is a nationwide organ-specific cancer registry for bone tumors.

## 2. Materials and Methods

### 2.1. Data Source

For this study, we used the Bone Tumor Registry in Japan, which is a nationwide organ-specific cancer registry for bone tumors that was started in the 1950s by the Japanese Orthopaedic Association (JOA). The basic data for the patients who were newly diagnosed at the participating hospitals were retrospectively collected annually. The basic data included patients’ characteristics, information on the tumor, and treatment received. The follow-up data for the patients whose basic data were registered were retrospectively collected at 2, 5, and 10 years after diagnosis. The follow-up data included the oncological outcome at the time of the latest follow-up. Although the interval of the follow-up was not standardized in all hospitals, the computer tomography of the chest was generally conducted every 3 to 6 months to evaluate lung metastasis in most hospitals. The Institutional Review Board (IRB) of the JOA approved this study protocol. Informed consent was not mandated by the Ethics Guidelines for Human Subject Medical Research and was waived by the IRB because the database was de-identified. We obtained data from the Bone Tumor Registry on high-grade osteosarcoma arising in the femur and pelvic bone that was diagnosed between 2006 and 2015. We excluded low-grade osteosarcomas such as periosteal and parosteal osteosarcoma. For each patient, we extracted the following data: date of diagnosis, sex, age at diagnosis, tumor size (maximum tumor diameter), primary or secondary osteosarcoma, presence of metastasis at diagnosis, surgical resection, and chemotherapy information, and oncological outcome at the last follow-up. Cases with insufficient data were excluded. Secondary osteosarcoma was defined as osteosarcoma arising after radiotherapy for other cancers, or arising in prior benign bone disease including Paget disease, fibrous dysplasia, or bone infarction. The drug used in osteosarcoma was almost standardized in all centers in Japan for young localized osteosarcoma. Most centers used, doxorubicin, cisplatin, methotrexate, and some centers added ifosfamide to them. However, for elderly osteosarcomas, metastatic osteosarcoma or palliative cases, not only those drugs but also various drugs were used such as gemcitabine, docetaxel, etoposide, or carboplatin. Chemotherapy was not given to the patients who were not tolerable to chemotherapy due to comorbidity or old age. Surgical resection was performed when the surgeon judged wide curative resection was possible. The possible reason for non-surgical treatment included that the surgeon judged they could not resect the tumor with a wide margin, for example, the tumor invading beyond the center of sacrum, that the patient refused aggressive surgery such as external hemipelvectomy, or that the patients could not be cured with surgical resection because uncontrolled metastases existed. For non-surgical cases, carbon ion radiotherapy was sometimes performed as a local curative treatment.

### 2.2. Statistical Analyses

The clinical characteristics of patients with femoral and pelvic osteosarcoma were compared using chi-square test for categorical variables and the Wilcoxon rank-sum test for continuous variables. Overall survival was defined as the period between the date of diagnosis and death. The Kaplan–Meier method was used for overall survival. The factors associated with survival were analyzed using the log-rank test for univariate analysis and the Cox proportional hazards model for multivariate analysis. The propensity score was defined as the probability of undergoing surgical resection of the primary tumor based on the baseline covariates. The propensity score was predicted from a multivariable logistic regression model. Variables for inclusion in the logistic model were selected based on factors associated with the likelihood of undergoing surgical resection of pelvic osteosarcoma, including sex, age at diagnosis, tumor size, primary or secondary osteosarcoma, presence of metastasis at diagnosis, and chemotherapy treatment. Inverse probability of treatment weighting (IPTW) was defined as the inverse of the probability of receiving the treatment that the patient received. All statistical analyses were conducted in R 3.1.1. The threshold for statistical significance was *p* < 0.05.

## 3. Results

### 3.1. Patient and Treatment Demographics for Femoral and Pelvic Osteosarcoma

A total of 632 patients with femoral osteosarcoma and 150 patients with pelvic osteosarcoma that were registered in the Bone Tumor Registry in Japan between 2006 and 2015 were enrolled in this study. Five radiation-induced osteosarcomas were included in 150 pelvic osteosarcomas. Mean follow-up time was 26 (1–85) months. The portion of patients with follow-up less than 6 months was 51/782 (6.5%). Patient demographics and treatments received are shown in Table 1. Pelvic osteosarcomas were more common in the elderly, had metastases at presentation, had larger tumor size, and were more likely to be secondary osteosarcomas compared to femoral osteosarcomas. In addition, the percentage of patients who did not undergo chemotherapy or surgical resection was higher in patients with pelvic osteosarcoma than in those with femoral osteosarcoma. The number of cases with wide, marginal, or intralesional resection was 545, 13, 11 in femoral osteosarcoma and 44, 5, 4 in pelvic osteosarcoma, respectively.

### 3.2. Overall Survival of Patients with Femoral and Pelvic Osteosarcoma

**Figure 1:**
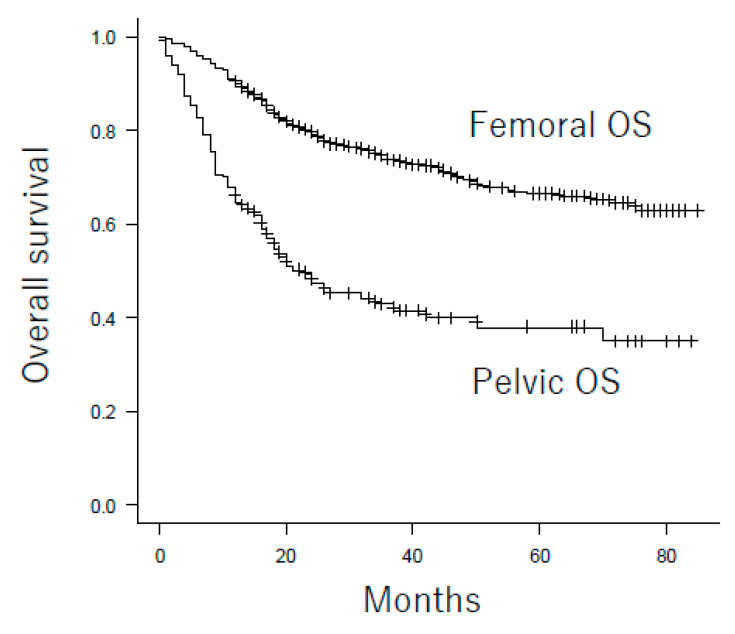
Overall survival for patients with pelvic osteosarcoma and femoral osteosarcoma. The 3-year overall survival rates were 42.8% in pelvic osteosarcoma and 73.7% in femoral osteosarcoma (*p* < 0.001). OS: osteosarcoma.

**Figure 2:**
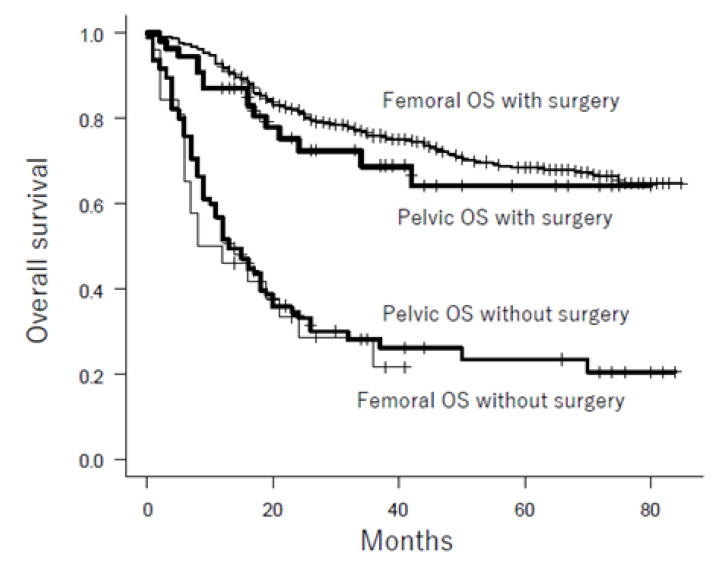
Subgroup analysis of pelvic osteosarcoma and femoral osteosarcoma according to surgical resection. There was no significant difference in overall survival between pelvic osteosarcoma and femoral osteosarcoma in patients who underwent surgical resection (*p* = 0.329) and in patients who did not undergo surgical resection (*p* = 0.644). OS: oseteosarcoma.

The overall survival rates of patients with femoral and pelvic osteosarcoma are shown in Table 2 and Figure 1. Patients with pelvic osteosarcoma had a significantly poorer prognosis than those with femoral osteosarcoma, with a 3-year survival rate of 42.8% and 73.7%, respectively. Other poor prognostic factors were also identified, including metastasis at presentation, advanced age, and large tumor size. In addition, patients who did not undergo chemotherapy or surgical resection had a poorer prognosis than the others, when the overall survival of all patients was examined. In the multivariate Cox hazards model for overall survival of all patients, metastasis at presentation, advanced age, large tumor size, treatment without chemotherapy, and treatment without surgical resection were significant poor prognostic factors (Table 3). Pelvic location was not, however, a significant prognostic factor. A subgroup analysis of overall survival for each category to compare pelvic osteosarcoma with femoral osteosarcoma was performed. Patients with pelvic osteosarcoma had a poorer prognosis than those with femoral osteosarcoma, regardless of gender, metastasis status at presentation, age at diagnosis, tumor size, and treatment with/without chemotherapy (Table 2). On the contrary, there was no significant difference in overall survival between pelvic osteosarcoma and femoral osteosarcoma in patients who underwent surgical resection and patients who did not undergo surgical resection (Figure 2). As such, we hypothesized that pelvic osteosarcoma had a poor prognosis because of the high percentage of patients who did not undergo surgical resection due to metastasis at presentation, large tumor size, or advanced age at diagnosis.

### 3.3. Characteristics of Pelvic Osteosarcoma Patients Treated with or without Surgical Resection

To investigate this hypothesis, we compared the characteristics of patients who underwent surgical resection of pelvic osteosarcoma and those who did not (Table 4). There were significantly more cases with metastases at presentation, cases of patients who were over 60 years old at the time of diagnosis, and cases with a tumor size of over 12 cm in the no resection group. We developed a logistic regression model to identify the factors that were significantly associated with surgical resection of pelvic osteosarcoma (Table 5). Patients with metastasis at presentation and patients who were more than 60 years old at the time of diagnosis had a significantly lower likelihood of surgical resection. Patients with a tumor size of more than 12 cm tended to be treated without surgical resection.

#### 3.3.1. Propensity Score Analysis of Surgical Resection of Pelvic Osteosarcoma

**Figure 3:**
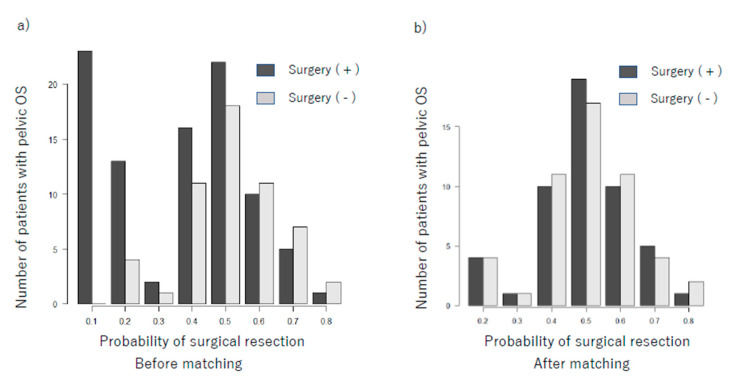
The imbalances of the propensity score (defined as the probability of undergoing surgical resection of the primary tumor in pelvic osteosarcoma) were diminished after propensity score matching. (**a**) Before propensity score matching (**b**) after propensity score matching.

**Figure 4:**
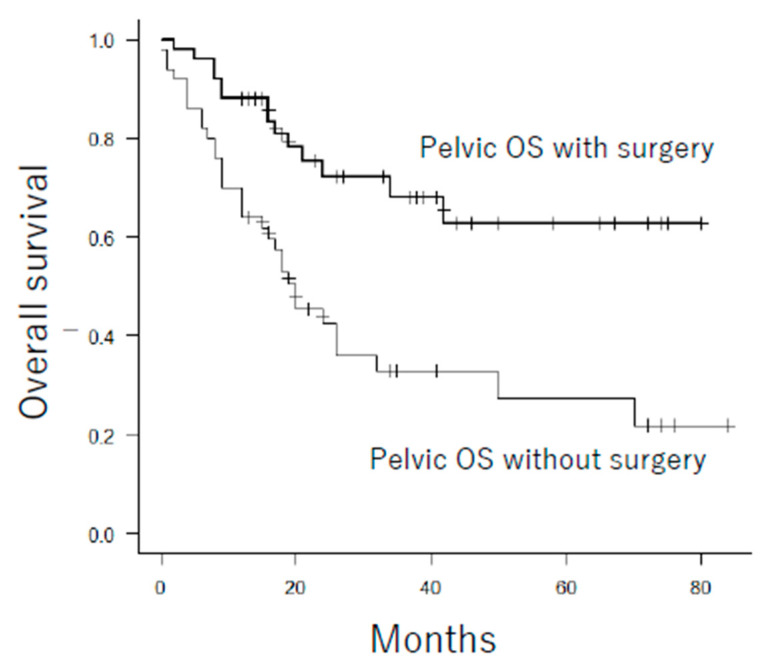
Overall survival for patients with pelvic osteosarcoma treated with and without surgical resection after propensity score matching (*p* < 0.001). OS: osteosarcoma.

Patients treated with surgical resection had a significantly better prognosis compared with patients treated without surgical resection. There were several differences between the two groups, however, as discussed. To reduce selection biases, propensity score analysis was performed. Various factors (as described in the Methods section) were used in the calculation of the propensity score and corresponding matching. Before matching, there were several differences between the two groups. After matching, the imbalances of these factors diminished. The distribution of the propensity scores showed a good overlap between the two groups (Table 4 and Figure 3). Even after propensity score matching, patients treated with surgical resection had a significantly better prognosis than those treated without surgical resection (Figure 4). We also performed propensity score analysis with IPTW. After IPTW analysis, patients who did not undergo surgical resection had a 2.7-fold greater hazard of death compared with patients who did (hazard ratio [HR] = 0.37, 95% confidence interval [CI]: 0.19–0.73, *p* = 0.004). Thus, surgical resection of pelvic osteosarcoma offers a survival benefit. Against this background, we concluded that pelvic osteosarcoma had a poor prognosis because it frequently occurred in the elderly, often had a larger tumor size, and had metastasis at presentation more often in comparison to femoral osteosarcoma. These three factors were also associated with non-surgical treatment, which also led to a poor outcome.

#### 3.3.2. Indication for Surgical Resection of Pelvic Osteosarcoma

**Figure 5:**
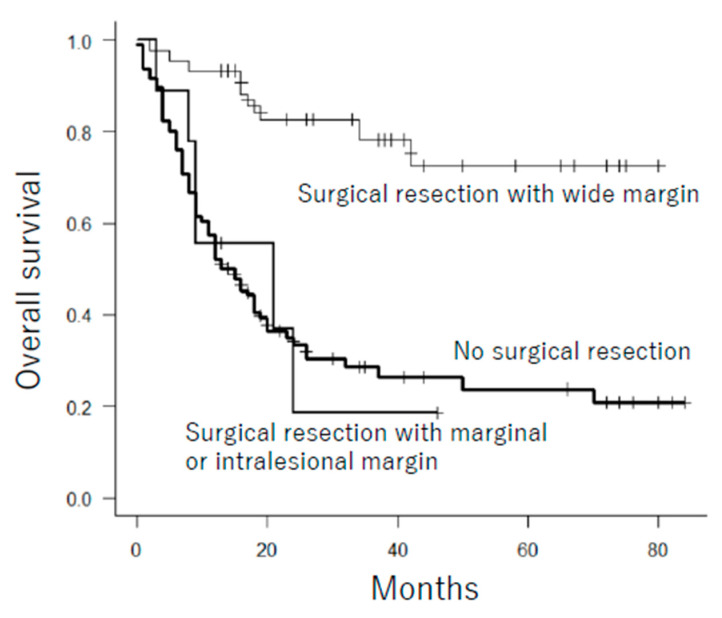
Overall survival for patients with pelvic osteosarcoma according to surgical margin. Patients who underwent wide resection had a significantly better prognosis than patients who underwent marginal or intralesional resection and patients treated without surgical resection (*p* < 0.001).

Next, we attempted to clarify the limitations of surgery for pelvic osteosarcoma. We classified all cases of pelvic osteosarcoma into four categories according to presence of metastasis and age at diagnosis (under or over 60 years old) which were the most negative prognostic factors of pelvic osteosarcoma. We indicated that overall survival was significantly improved by surgical resection in young localized patients (Table 6). For elderly localized patients and young metastatic patients, surgical resection tended to improve overall survival but not significantly. No cases over 60 years old with metastasis at presentation underwent surgical resection of pelvic osteosarcoma. In addition, there was no difference in prognosis between patients who underwent marginal or intralesional resection and patients treated without surgical resection (Figure 5). Patients who underwent wide resection (*n* = 45) had a significantly better prognosis than patients treated with marginal or intralesional resection (*n* = 9) and without surgical resection (*n* = 95) (*p* < 0.001). Therefore, surgical resection for pelvic osteosarcoma should be selected in patients whose tumor can be resected with a wide margin.

## 4. Discussion

Although pelvic osteosarcomas have been reported to have a poorer prognosis than osteosarcomas arising in other locations [2,9], the reasons for this remain unknown. Here, we demonstrated that the poor prognosis of osteosarcoma resulted from the fact that a high percentage of patients with pelvic osteosarcoma were elderly at diagnosis, had metastasis at presentation, and had a large tumor at presentation. Therefore, the high percentage of pelvic osteosarcoma patients that did not undergo surgical resection was associated with a poor prognosis. We also demonstrated that surgical resection improves the prognosis of pelvic sarcoma by propensity score analysis. Moreover, we suggested that surgical resection of pelvic osteosarcoma should be considered based on the stage at diagnosis and age at presentation. The surgical resection should always be performed with a wide margin.

### 4.1. Reasons for the Poor Prognosis of Pelvic Osteosarcoma Based on the Comparison of Pelvic and Femoral Osteosarcoma

Pelvic osteosarcoma occurred frequently in the elderly, often had a larger tumor size, and more frequently had metastasis at presentation in comparison to femoral osteosarcoma. These three factors were associated with a poor prognosis of pelvic osteosarcoma. These factors were also associated with no surgical treatment, which led to a poor outcome. Pelvic osteosarcoma is likely to have delayed diagnosis and to thus have larger tumor size and metastasis before diagnosis. A large tumor size makes it difficult to achieve tumor resection with a wide margin and leads to a poor outcome. In addition, sufficient chemotherapy cannot be given to elderly patients, which may also lead to a poor outcome. In the surgical cases only, however, there was no difference in the prognosis between pelvic osteosarcoma and femoral osteosarcoma, unlike in previous studies [13]. A previous report indicated that pelvic osteosarcoma had a poor prognosis even after surgical resection. This discrepancy may be related to the fact that secondary osteosarcoma from Paget’s disease is rare in Japan, or to improvements in surgical technique that reduced the number of inadequate resections, or to the limited number of patients who underwent surgical resection. In this study, secondary osteosarcoma of pelvic osteosarcomas accounted for 1.8% of cases, although in previous reports from Western countries, it accounted for 23 to 38% of cases [9,13]. A wide margin was achieved in 43 (82.7%) of 52 cases treated with surgical resection in this study and was previously reported to be 69% [13]. In this study, surgical cases were limited to 36.7% of all pelvic osteosarcoma, and this was previously reported to be 67 to 75% [5,7,9]. The high rate of non-resected tumors might influence the results of our study. The high rate of non-resected tumors in this study may be related to the introduction of carbon ion radiotherapy for unresectable sarcoma in Japan. Carbon ion radiotherapy for unresectable osteosarcoma has been reported to have a 33% 5-year survival rate [14]. It is a feasible surgical alternative to unresectable pelvic osteosarcoma. However, this study showed that the young localized pelvic osteosarcoma at least should receive the surgical resection because their prognosis will be improved by surgical resection.

### 4.2. Impact and Indication of Surgical Resection of Pelvic Osteosarcoma

Many reports have indicated the importance of surgical resection of osteosarcoma [9,15]. As most of these studies are retrospective, however, the effects of uncontrolled bias cannot be ruled out. We indicated that surgical resection of pelvic osteosarcoma may improve the outcome by adjusting the confounding factors using propensity score analysis. We could not obtain the precise reason why the surgery was not performed from the data in this database. However, the analysis of the probability of undergoing surgical resection showed that patients with metastasis at presentation and patients who were more than 60 years old at the time of diagnosis had a significantly lower likelihood of surgical resection, and patients with a tumor size of more than 12 cm tended to be treated without surgical resection (Table 5). Subgroup analysis revealed that for young patients without metastasis, surgical resection significantly improved the outcome. As such, surgical resection should be performed in these patients without compromises to achieve the wide resection even if external hemipelvectomy is required.

Controversial surgical indications include localized cases in elderly patients and metastatic cases in young patients. Elderly patients with osteosarcoma have a poorer prognosis than young patients as they may not be able to tolerate the aggressive chemotherapy that is attributed to improving the prognosis of osteosarcoma in young patients. There is no clear evidence of the effectiveness of chemotherapy for elderly osteosarcoma [3,15,16,17,18]. However, it is controversial how old patients should be considered in elderly osteosarcoma. Some studies, including groups aged 40–60 years as elderly osteosarcoma reported chemotherapy improved survival [19,20]. In this study, 60 years old was set as cut-off point because the elderly of more than 60 years old may not be able to tolerate the intensive planned chemotherapy. Many studies have shown that definitive surgical resection is a significant prognostic factor for osteosarcoma in elderly patients [15,16,17], although it is not significant in this study. Thus, surgical resection before chemotherapy is one of the options for the elderly localized cases where postoperative function seems to be maintained, for example, cases not involving the acetabulum. It could reduce the complication rate. On the other hand, we also showed that the prognosis remained poor even after surgical resection for pelvic osteosarcoma in elderly patients (3-year overall survival rate of 39%). Of the 15 patients who underwent surgical resection for localized pelvic osteosarcoma in the elderly, 12 patients (80%) were resected with a wide margin. Of the 36 patients who underwent surgical resection for young localized pelvic osteosarcoma, 29 patients (81%) were resected with a wide margin. The percentage of the patients who underwent surgical resection with a wide margin was similar. Thus, the poor prognosis in elderly pelvic osteosarcoma was not due to the surgical margin but to the nature of the tumor itself, insufficient chemotherapy, or the frailty of the elderly. Additionally, it has been reported that dysfunction after surgical resection for pelvic osteosarcoma involving the acetabulum, especially in elderly patients, is severe [21].

### 4.3. Additional/Other Treatment Options

Instead of surgical resection, we think carbon ion radiotherapy could be the alternative treatment option for the cases in the elderly pelvic osteosarcoma where postoperative dysfunction seems to be very severe. Matsunobu et al. reported on carbon ion radiotherapy for unresectable osteosarcoma in elderly patients with a 34% 5-year overall survival rate [14], which was comparable to the result of surgical cases in this study. Thus, carbon ion radiotherapy may be a feasible alternative treatment to surgical resection for pelvic osteosarcoma in elderly patients, although it also causes complications and dysfunctions [22]. Clinical studies with time trade-off analyses comparing life expectancy and functional impairment after surgical resection or carbon ion radiotherapy are needed in order to identify the optimal treatment for elderly patients with localized pelvic osteosarcoma.

The local tumor resection between the preoperative and postoperative chemotherapy has become the standard protocol worldwide for localized juvenile osteosarcoma, although there is no clear evidence of the effectiveness of adding preoperative chemotherapy in postoperative chemotherapy because preoperative chemotherapy may shrink the tumor and allow safer and more functional surgery. On the other hand, a standard treatment for metastatic osteosarcoma has not been established. The main treatment for metastatic osteosarcoma is chemotherapy. We believe that surgical resection for the primary tumor is not indicated in elderly patients with metastatic disease who are unable to undergo sufficient chemotherapy. It has been reported that complete surgical resection of primary and metastatic lesions with aggressive chemotherapy has prognostic value for primary metasatatic osteosarcoma in young patients [23]. Thus, when chemotherapy is effective and all metastatic lesions can be resected, the resection of the primary lesion may make sense even in patients with metastasis at presentation. However, surgery for pelvic osteosarcoma causes dysfunction and involves numerous complications, including infection, which sometimes make postoperative chemotherapy impossible [24]. Thus, we suggest that surgical resection of the primary site for metastatic pelvic osteosarcoma be performed at the end of the treatment (after all chemotherapy and local treatment for metastases), while it needs to be verified.

### 4.4. Future Direction

In previous reports, we indicated that surgical resection with a wide margin was a significant prognostic factor. Pelvic tumors are difficult to remove with an adequate margin, however. In order to secure a wide margin, the use of surgical assistant technologies, such as navigation, three-dimensional printing models, and custom-made cutting guide, are recommended [25,26,27]. There was no paper discussing the difference in genetic abnormality of osteosarcoma depending on the site of primary. However, it has been reported that tumor mutation burden was significantly higher in elderly osteosarcoma than in younger osteosarcoma [28]. Because pelvic osteosarcoma is more common in the elderly, we think there is a genetic difference between pelvic and femoral osteosarcoma. Basic research on osteosarcoma, including genomic analysis, will provide new perspectives and therapeutic agents to treat this challenging disease.

### 4.5. Limitations

There were some limitations to this study because it was retrospective and based on registry data. The extension of the tumor, the comorbidity, or detailed chemotherapy data, such as protocol and dose, were not available from the Bone Tumor Registry in Japan. As there were many cases in which the histological subtype was not registered, the difference in histological subtype between pelvic osteosarcoma and femoral osteosarcoma could not be analyzed. The follow-up time was too short to analyze 5 year survival. Patient registration is not mandatory for non-JOA-certified hospitals, and there is a possibility that some patients were treated at non-specialized hospitals. We expect that the majority of osteosarcomas would have been treated at specialist centers certified by the JOA, however.

## 5. Conclusions

The reasons for the poor prognosis of pelvic osteosarcoma are that it is common in the elderly, that there are many cases of metastatic disease at presentation, that there are many cases with large tumor size, and that surgical resection is seldom performed because of these factors. Surgical resection with an adequate margin improves the prognosis of pelvic osteosarcoma. Therefore, it should be performed for young patients with localized pelvic osteosarcoma where the surgeon judged the wide margin cannot be secured, and it should also be considered as a treatment option for elderly patients without metastasis and young patients with metastasis. Further research is needed to identify the optimal treatment for these patients.

## Figures and Tables

**Table 1:** Clinical characteristics of the patients with femoral and pelvic osteosarcoma.

Variables		Patient Number (%)		*p*
		Femoral OS	Pelvic OS	
Gender	Male	338 (53.5%)	73 (48.7%)	0.332
	Female	294 (46.5%)	77 (51.3%)	
Metastasis at presentation	M0	531 (84.7%)	109 (73.2%)	**0.001**
	M1	96 (15.3%)	40 (26.8%)	
Age	mean	27.58 years	50.63 years	**<0.001**
	<40 years	473 (74.8%)	45 (30.0%)	**<0.001**
	≥40 years	159 (25.2%)	105 (70.0%)	
	<60 years	542 (80.1%)	90 (60.0%)	**<0.001**
	≥60 years	90 (14.2%)	60 (40.0%)	
Size	mean	10.63 cm	11.12 cm	0.278
	<8 cm	163 (27.0%)	32 (21.9%)	0.247
	≥8 cm	440 (73.0%)	114 (78.1%)	
	<12 cm	408 (67.7%)	84 (57.5%)	**0.027**
	≥12 cm	195 (32.3%)	62 (42.5%)	
Secondary	Primary	624 (98.7%)	144 (96.0%)	0.054
	Secondary	8 (1.3%)	6 (4.0%)	
Chemotherapy	No	71 (11.3%)	31 (20.7%)	**0.003**
	Yes	560 (88.7%)	119 (79.3%)	
Surgical resection	No	26 (4.1%)	95 (63.3%)	**<0.001**
	Yes	606 (95.9%)	55 (36.7%)	

OS: osteosarcoma.

**Table 2:** Overall survival rate at 3 years in all cases, femoral osteosarcoma, and pelvic oseteosarcoma.

		3-Year OS Rate		3-Year OS Rate		
Variables		All Cases	*p*	Femoral OS	Pelvic OS	*p*
Location	Femoral	73.7%	**<0.001**			
	Pelvis	42.8%				
Gender	Male	68.3%	0.818	72.9%	45.7%	**<0.001**
	Female	67.4%		74.6%	39.0%	**<0.001**
Metastasis at presentation	M0	75.8%	**<0.001**	80.6%	52.0%	**<0.001**
	M1	29.3%		33.9%	19.7%	**0.002**
Age at diagnosis	<40 years	77.9%	**<0.001**	79.9%	55.7%	**<0.001**
	≥40 years	48.5%		55.8%	37.3%	**<0.001**
	<60 years	74.2%	**<0.001**	76.6%	59.5%	**<0.001**
	≥60 years	41.8%		56.9%	19.0%	**<0.001**
Size	<12 cm	73.2%	**<0.001**	77.6%	51.5%	**<0.001**
	≥12 cm	56.7%		64.4%	32.2%	**<0.001**
Secondary	Primary	68.3%	0.085	73.8%	43.5%	**<0.001**
	Secondary	48.2%		62.5%	25.0%	0.163
Chemotherapy	Yes	70.2%	**<0.001**	74.9%	48.0%	**<0.001**
	No	52.1%		64.0%	22.4%	**<0.001**
Surgical resection	Yes	75.5%	**<0.001**	76.0%	68.9%	0.329
	No	26.6%		21.6%	28.2%	0.644

OS: osteosarcoma.

**Table 3:** Multivariate Cox Hazard models for overall survival of the patients with femoral and pelvic osteosarcoma.

Variables	HR	95% CI	*p*
Location: pelvis	1.01	0.67–1.51	0.976
Gender: female	1.06	0.82–1.36	0.679
Metastasis at presentation: yes	3.56	2.62–4.84	**<0.001**
Age ≥60 years	2.96	2.15–4.06	**<0.001**
Size ≥12 cm	1.38	1.05–1.81	**0.021**
Secondary osteosarcoma	0.96	0.42–2.21	0.919
Chemotherapy: yes	0.92	0.63–1.34	0.661
Surgical resection: yes	0.35	0.23–0.53	**<0.001**

HR: hazard ratio, CI: confidence interval.

**Table 4:** Characteristics of the pelvic osteosarcoma treated with or without surgical resection before and after propensity score matching.

		Patient Number (%) (*n* = 150)			Patient Number (%) after Propensity Score Matching (*n* = 92)		
Variables		Resection	No Resection	*p*	Resection	No Resection	*p*
Gender	Male	26 (47.3%)	47 (49.5%)	0.928	22 (47.8%)	22 (47.8%)	1.00
	Female	29 (52.7%)	48 (50.5%)		24 (52.2%)	24 (52.2%)	
Metastasis at presentation	M0	51 (92.7%)	58 (61.7%)	**<0.001**	42 (91.3%)	43 (93.5%)	1.00
	M1	4 (7.3%)	36 (38.3%)		4 (8.7%)	3 (6.5%)	
Age	<40 years	19 (34.5%)	26 (27.4%)	0.460	17 (37.0%)	17 (37.0%)	1.00
	≥40 years	36 (65.5%)	69 (72.6%)		29 (63.0%)	29 (63.0%)	
	<60 years	40 (72.7%)	50 (52.6%)	**0.025**	32 (69.6%)	32 (69.6%)	1.00
	≥60 years	15 (27.3%)	45 (47.4%)		14 (30.4%)	14 (30.4%)	
Size	<12 cm	40 (74.1%)	44 (47.8%)	**0.003**	33 (71.7%)	31 (67.4%)	0.82
	≥12 cm	14 (25.9%)	48 (52.2%)		13 (28.3%)	15 (32.6%)	
Secondary	Primary	52 (94.5%)	92 (96.8%)	0.795	45 (97.8%)	45 (97.8%)	1.00
	Secondary	3 (5.5%)	3 (3.2%)		1 (2.2%)	1 (2.2%)	
Chemotherapy	Yes	44 (80.0%)	75 (78.9%)	1	39 (84.8%)	40 (93.5%)	1.00
	No	11 (20.0%)	20 (21.1%)		7 (15.2%)	6 (13.0%)	

**Table 5:** Logistic regression model for the probability of undergoing surgical resection of pelvic osteosarcoma.

Variables	Odds Ratio	95% CI	*p*
Gender: female	1.24	0.57–2.69	0.583
Metastasis at presentation: yes	0.167	0.052–0.53	**0.002**
Age ≥60 years	0.299	0.12–0.75	**0.010**
Size ≥12 cm	0.46	0.20–1.05	0.065
Secondary osteosarcoma	3.25	0.46–23.0	0.238
Chemotherapy: yes	0.754	0.26–2.19	0.605

**Table 6:** Subgroup analysis of pelvic osteosarcoma according to the stage, age, and surgical resection.

Stage at Presentation	Age at Diagnosis	Surgical Resection	No. of Patients	3-Year OS Rate	*p*
Localized	<60 years old	Yes	36	83.0	**0.005**
Localized	<60 years old	No	32	50.9	
Localized	≥60 years old	Yes	15	39.1	0.117
Localized	≥60 years old	No	26	22.0	
Metastatic	<60 years old	Yes	4	75.0	0.251
Metastatic	<60 years old	No	18	29.2	
Metastatic	≥60 years old	Yes	0	N.A	N.A.
Metastatic	≥60 years old	No	18	0.0	

## Data Availability

The data presented in this study are available on request from the corresponding author.
